# Processes of Electrospun Polyvinylidene Fluoride-Based Nanofibers, Their Piezoelectric Properties, and Several Fantastic Applications

**DOI:** 10.3390/polym14204311

**Published:** 2022-10-13

**Authors:** Yubin Bai, Yanan Liu, He Lv, Hongpu Shi, Wen Zhou, Yang Liu, Deng-Guang Yu

**Affiliations:** 1 School of Materials and Chemistry, University of Shanghai for Science and Technology, Shanghai 200093, China; 2 School of Chemistry and Chemical Engineering, Shanghai University of Engineering Science, 333 Long Teng Road, Shanghai 201620, China

**Keywords:** electrospinning, polyvinylidene fluoride, piezoelectric properties, biomedicines, energy, photocatalysis

## Abstract

Since the third scientific and technological revolution, electronic information technology has developed rapidly, and piezoelectric materials that can convert mechanical energy into electrical energy have become a research hotspot. Among them, piezoelectric polymers are widely used in various fields such as water treatment, biomedicine, and flexible sensors due to their good flexibility and weak toxicity. However, compared with ceramic piezoelectric materials, the piezoelectric properties of polymers are poor, so it is very important to improve the piezoelectric properties of polymers. Electrospinning technology can improve the piezoelectric properties of piezoelectric polymers by adjusting electrospinning parameters to control the piezoelectrically active phase transition of polymers. In addition, the prepared nanofibrous membrane is also a good substrate for supporting piezoelectric functional particles, which can also effectively improve the piezoelectric properties of polymers by doping particles. This paper reviews the piezoelectric properties of various electrospun piezoelectric polymer membranes, especially polyvinylidene fluoride (PVDF)-based electrospun nanofibrous membranes (NFs). Additionally, this paper introduces the various methods for increasing piezoelectric properties from the perspective of structure and species. Finally, the applications of NFs in the fields of biology, energy, and photocatalysis are discussed, and the future research directions and development are prospected.

## 1. Introduction

**Figure 1:**
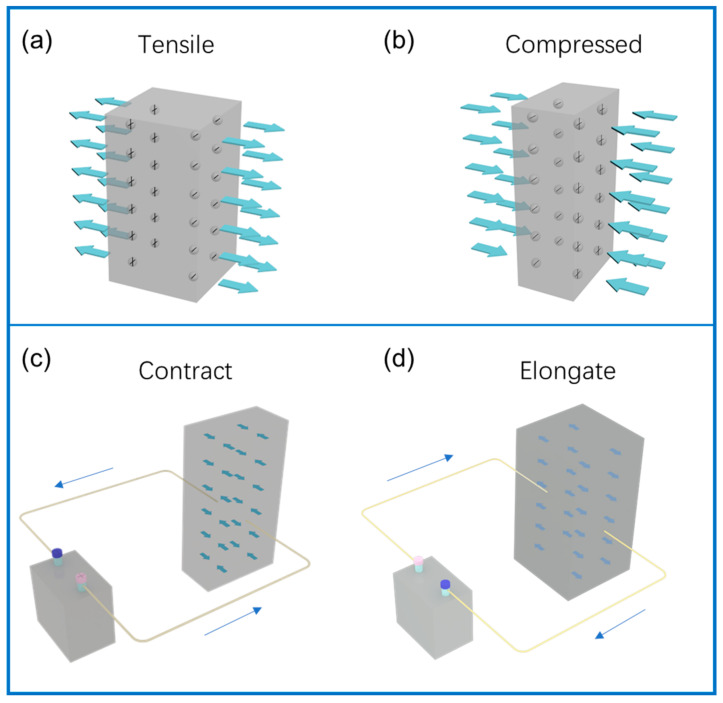
Schematic diagram of positive piezoelectric effect and negative piezoelectric effect. (**a**,**b**) show the positive piezoelectric effect exerted by compressive and tensile forces, respectively, the arrows represent tensile and compressive forces. (**c**,**d**) represent the negative piezoelectric effect, which causes the piezoelectric material to shrink and elongate, respectively, the arrow represents the direction of the current.

With the continuous progress and development of science and technology, piezoelectric materials can play an important role in the mutual conversion between mechanical force and electrical signals, and these materials have gradually attracted people’s attention [1]. In 1880, P. Curie and J. Curie discovered the piezoelectric effect in quartz crystals [2]; the piezoelectric effect is divided into positive and negative piezoelectric effects. When a mechanical force is applied in a direction and deformed, polarization occurs inside, and opposite positive and negative charges are generated on the two opposite surfaces; a piezoelectric material can generate a built-in electric field under the action of mechanical stress. After removing the external force, the positive and negative charges disappear and return to the original state. On the contrary, there is also an inverse piezoelectric effect: the application of an external electric field can cause deformation of the material. After the electric field is removed, the deformation of the dielectric disappears, as shown in Figure 1. According to the principle of the piezoelectric effect, the material is able to convert the information of the structural deformation into an electrical signal [3]. This potential change can be captured by external detection equipment, which can be applied to energy harvesters [4,5], ultrasonic transducers [6,7], sensors [8], biomedicine [9,10,11], and nanogenerators [12,13]. Piezoelectric effects can also be used as a means of separating charges in photocatalysis [14].

Piezoelectric materials can be divided into ceramic material and polymer material categories. Many lead-based and non-lead-based ceramic piezoelectric materials have been reported, such as lead metaniobate (PbNb_2_O_6_) [15], lead titanate (PbTiO_3_) [16], barium titanate (BaTiO_3_) [17,18], barium lead niobate (PBLN) [19], lithium tetraborate (Li_2_B_4_O_7_) [20], lead zirconate titanate (PZT) [21], zinc oxide (ZnO) [22], and others. Although these piezoelectric materials have good piezoelectric properties, the flexibility of such materials is generally poor, which limits their application in flexible pressure sensors. PVDF is a semi-crystalline polymer composed of [CH_2_=CF_2_] monomer polymerization, its piezoelectric constant (20–28 pC/N) is the best among polymers [23], and it possesses good flexibility, piezoelectric properties, a dielectric constant, pyroelectric effect, ferroelectric effect, biocompatibility, and chemical stability [24]. There are five polymorphs of PVDF produced by different processing conditions, including α and δ (TGTG’), β (TTTT), and γ and ε (TTTGTTTG’) phases [25]. Among them, α and ε phases are non-polar phases, while the β, γ, and δ phases are polar phases. The α phase is the most common phase in nature, but the β phase of PVDF exhibits the best piezoelectric properties. The melt crystallization of PVDF produces α phase [26,27,28,29]. In order to obtain better piezoelectric materials, β phase with good piezoelectric properties is needed.

Generally, the methods of converting the non-polar α phase in PVDF to the polar β phase include heat treatment [30], mechanical stretching [31], high-voltage electric field application [32], electrospinning [33,34,35], functional particles addition [36,37,38], and others. Compared with other methods, electrospinning is a simple and low-cost technology, which can produce fibers with diameters from micro-size to nano-size [39,40,41], and it can effectively convert the α phase of PVDF into β phase. Additionally, various functional particles are easily incorporated into the nanofibers by electrospinning, and functional particles/PVDF composite nanofibrous membranes with improved piezoelectric properties are easily constructed. NFs have many advantages; the piezoelectric constants of inorganic and polymeric piezoelectric materials are listed in Table 1. The piezoelectric properties of PBLN are the best among ceramic materials, while the piezoelectric properties of PVDF NFs are the best among polymers. The piezoelectric properties of polyacrylonitrile (PAN) and cellulose NFs are not excellent, but the good piezoelectric properties of composite fiber membranes can be obtained by adding other piezoelectric materials. Thus, these polymers are often used as substrates for piezoelectric membrane materials.

This review starts from the research on the preparation of NF piezoelectric materials by electrospinning, and introduces the preparation methods of various piezoelectric NFs and their advantages and disadvantages, including PVDF NFs, PVDF organic blend NFs, doped functional particles, PVDF composite NFs, PVDF copolymer NFs, and other piezoelectric polymer NFs. The effects of the spinning process, addition of functional particles, and blending of components on the piezoelectric properties of materials—as well as the effect mechanism of the structure–activity relationship of materials on piezoelectric properties of materials—are mainly discussed. Finally, the applications of piezoelectric NFs in biomedicine, energy, and photocatalysis are introduced, and a summary and prospect are made.

## 2. Preparation of PVDF NFs by Electrospinning

### 2.1. Introduction of Electrospinning Principle and Parameters

**Figure 2:**
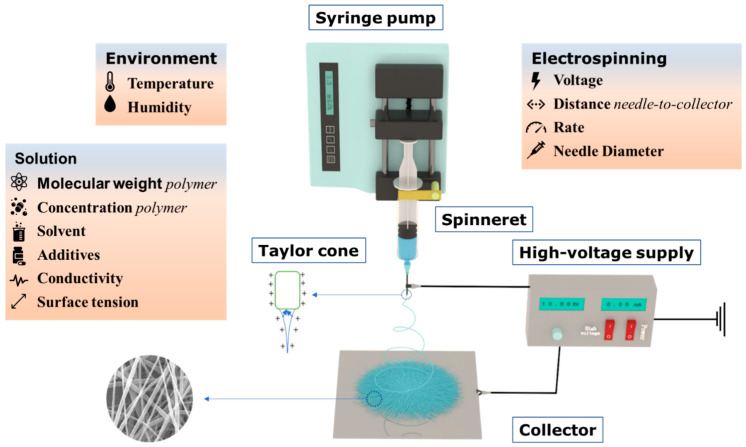
Electrospinning schematic diagram and influencing parameters.

The electrospinning device is generally composed of a high-voltage power supply, injection pump, spinning head, and receiving plate [69]. The basic principle of electrostatic spinning is the viscosity of the polymer solution through Coulomb force stretching, where spinning polymer droplets (due to Coulomb and gravity forces greater than the surface tension) gradually change from hemispherical to conical, and from a cone pointed outward to a liquid, and the solvents evaporate quickly, creating fine fibers in the receiving plate, as shown in Figure 2, eventually forming nanometer fiber membranes [70,71,72,73,74,75]. NFs have excellent properties such as small diameter, large porosity, large specific surface area, and good mechanical energy along the fiber axis; the fiber morphology can be controlled by adjusting the parameter settings [76,77]. The influencing factors can be divided into three categories, including electrospinning parameters (voltage, needle diameter, feed rate, distance from needle to collector), solution properties (polymer molecular weight, concentration, solvent, viscosity, conductivity, surface tension), and environment parameters (temperature and relative humidity). The influence of different electrospinning parameters on the morphology and piezoelectric properties of PVDF NFs are analyzed in following sections.

**Figure 3:**
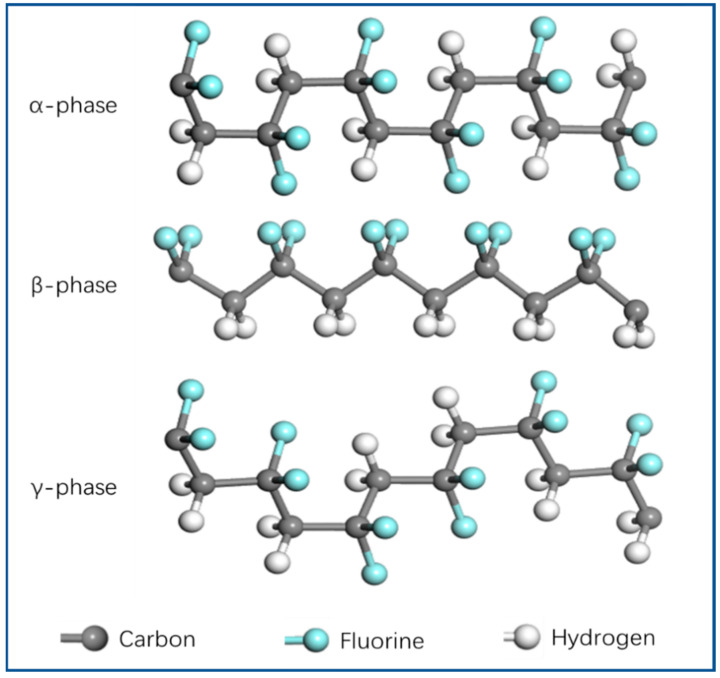
Molecular structure of the α, β, and γ phases of PVDF.

The piezoelectric properties of PVDF are related to its strong dipole moment. Since the electronegativity of fluorine atoms is greater than that of hydrogen and carbon atoms, PVDF exhibits a large dipole moment. When the PVDF monomer polymerizes to form a crystal and produces a dipole, there is a net dipole moment in the β phase where the fluorine atoms are arranged on one side of the polymer chain, and the net dipole moment disappears in the α phase where the fluorine atoms are evenly distributed on both sides of the chain. There is also a weak net dipole moment in the γ phase [78]. Therefore, the β phase with the highest piezoelectric properties has attracted much attention. Many methods have been developed to obtain high-β-phase content. The electrospinning method is a simple and effective method to obtain the β phase; PVDF nanofiber membranes prepared by electrospinning have a higher content of β phase. Due to the stretching of the jet and its own electrical polarization during spinning, the non-polar α phase and weakly polar γ phase are transformed into polar β phase, which has strong piezoelectric properties. The molecular chain conformation of α phase, β phase, and γ phase is shown in Figure 3.

### 2.2. Effect of Electrospinning Parameters on Piezoelectric Properties of Pure PVDF

The morphology and properties of NFs are mainly determined by the following three aspects: process parameters, the nature of the polymer solution, and environmental parameters [79,80,81,82,83]. The influence of three parameters on the morphology and piezoelectric properties of PVDF is introduced in the following section.

#### 2.2.1. Process Parameters

The process parameters included voltage, distance between receiving plate and spinning head, propulsion speed of syringe pump, and diameter of spinning head. During electrospinning, the effect of voltage on the structure and morphology of the fiber is enormous [84]; as the voltage increases, the diameter of the fiber decreases, and when the voltage increases to a certain value, the diameter of the fiber decreases and there is no more bead structure and no further increase in fiber diameter because the Taylor cone is smaller, and due to the increase in Coulomb force, the jet flow rate increases at the same propulsion speed [85]. 

**Figure 4:**
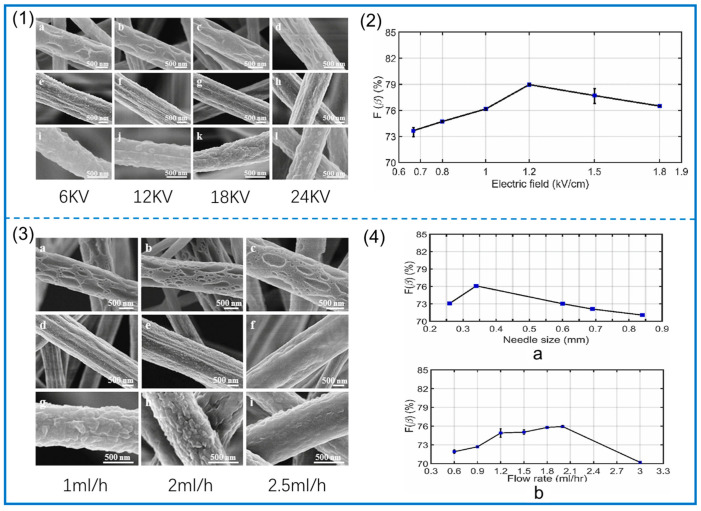
(**1**) SEM images of surface morphology of electrospun PVDF fibers under different applied voltage levels (**a**,**e**,**i**) 6 kV; (**b**,**f**,**j**) 12 kV; (**c**,**g**,**k**) 18 kV; (**d**,**h**,**l**) 24 kV. Reprinted with permission from Ref. [86], copyright 2019, IOP Publishing. (**2**) curves of percentage of β phase in PVDF NFs and voltage values of 10, 12, 15, 18, 22.5, and 27 kV. Reprinted with permission from Ref. [88], copyright 2021, Polymer. (**3**) SEM images of surface morphology of electrospun PVDF fibers at different flow rates (**a**,**d**,**g**) 1 mL/h; (**b**,**e**,**h**) 2 mL/h; (**c**,**f**,**i**) 2.5 mL/h. Reprinted with permission from Ref. [86], copyright 2019, IOP Publishing. (**4**) (**a**) Relationship between the proportion of β phase of PVDF NFs and the size of the needle eye (flow rate 2.0 mL/h). (**b**) Relationship between the percentage of β phase and the flow rate of solution (needle diameter 0.6 mm). Reprinted with permission from Ref. [88], copyright 2021, Polymer.

Zaarour et al. [86] studied the influence of process parameters on the surface morphology of PVDF. Dimethylformamide (DMF), acetone (ACE), and DMF/ACE were used as solvents at voltages of 6, 12, 18, and 24 kV, and other amounts were kept constant. Finally, the average diameter of the fibers was measured, and it was found that the diameter of the fibers gradually decreased when the voltage was gradually increased. When the voltage is changed from 18 kV to 24 kV, the fiber diameter increases. In addition, the surface morphology of the fibers is different. When the voltage gradually increases, the large surface aperture becomes elliptical and the grooves become more numerous because the Taylor cone is stretched more rapidly, and the roughness decreases because it is less affected by the buckling instability, as shown in Figure 4(1). In addition, the PVDF fiber under high voltage is more uniform, and the crystallinity of PVDF is improved [87]. In this process, the polymer solution is stretched to produce β phase due to the effect of the electric field. Singh et al. [88] studied the influence of process parameters on the formation of β phase in PVDF. Using DMF/ACE as a solvent, the voltage was determined as a single variable, and electrospinning was carried out at voltages of 10, 12, 15, 18, 22.5, and 27 kV. These voltage values corresponded to 0.67, 0.8, 1.0, 1.2, and 1.2 kV, respectively. Electric fields of 1.5 and 1.8 kV/cm were used. The β phase in the samples was determined by infrared spectroscopy, and it was concluded that the content of β phase in the fibers obtained from 10 kV to 18 kV voltage increased gradually (up to 79%), while the content of β phase in the fibers obtained from increasing the voltage continued to decrease, as shown in Figure 4(2). This is because with the continuous increase in voltage (greater than 18 kV), the flow rate also gradually increases, the evaporation time of the solvent is shortened, and the effective stretching of the NFs is reduced. The effect of voltage on the crystalline phase transition was also confirmed in the work of Nugraha et al. [87], and the change in β phase is a key factor affecting the piezoelectric properties of PVDF.

The distance between the receiving plate and the spinning head mainly affects the evaporation of the solvent, and because the distance affects the electric field strength, it affects the stretching of the polymer. Usually, this distance is set to 15 cm [89], and the larger the distance, the finer the diameter of the fiber [90,91]. When the spacing is shortened to very small, uneven fibers are obtained [92], and large-diameter fibers with many defects are produced [93]. The content of α phase decreases with a decrease in this distance, and the content of β phase increases with a decrease in this distance [94]. However, this parameter has little effect on the fiber [95]. Generally, the distance between the receiving plate and the spinning head often affects the fiber morphology and performance together with parameters such as voltage and flow rate.

The propulsion speed of the injection pump directly affects the flow rate of the polymer solution and maintaining a stable flow rate is a key factor in the production of homogeneous fibers; faster flow rates are not conducive to fiber formation and may result in coarse fibers or even unstable jets that can damage fiber formation [96]. Relatively coarse fibers may even produce unstable jet damage formation, which is unfavorable to solvent evaporation; velocities exceeding the critical value would produce microspheres [85]. However, the lower flow rate also affects the formation of Taylor cones. At a low flow rate, more elliptical large holes are formed on the fiber surface. At a high flow rate, the number of holes is reduced, and jet instability is weakened due to the increase in flow rate, resulting in a smoother surface, as shown in Figure 4(3). Flow rate also has a great influence on the formation of β phase. Singh et al. [88] studied the effect of flow rate change on the formation of β phase and found that the content of β phase also increased when the flow rate gradually increased from 0.6 mL/h to 2.0 mL/h; then, as they continued to increase the flow rate, the content of β phase began to decrease, which was when the flow rate was small. Because the shear force of the needle wall leads to the instability of the solution flow, the solution flow tends to be stable when the flow rate increases. However, when the flow rate continues to increase, the stretching of the fiber is incomplete due to the delayed evaporation of the solvent, and the content of β phase is reduced. The diameter of the spinning head can also have an effect on the formation of PVDF β phase, and a decrease in the diameter of the needle can change the number of solution droplets that flow out of the needle. When the diameter of the needle becomes smaller, the polymer solution drops from the needle shrink, and the increase in surface tension affects the jet, thus affecting surface morphology and piezoelectric properties, as shown in Figure 4(4).

#### 2.2.2. Polymer Solution Parameters

**Figure 5:**
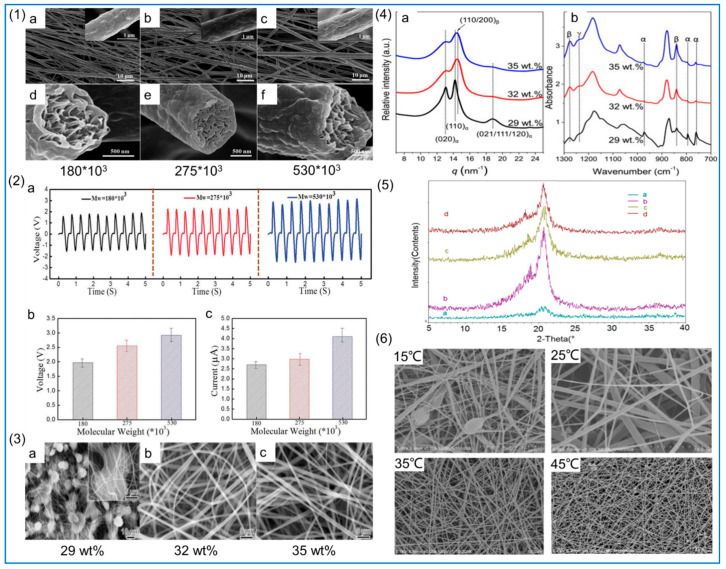
(**1**) SEM images of electrospun PVDF NFs with different molecular weights (MWs) (**a**,**d**) 180 × 10^3^; (**b**,**e**) 275 × 10^3^; (**c**,**f**) 530 × 10^3^. Reprinted with permission from Ref. [99], copyright 2019, Taylor & Francis. (**2**) Piezoelectric effect of PVDF NFs under different MWs. (**a**) Voltage output. (**b**) Statistical results of average voltage output. (**c**) Statistical results of average current output. Reprinted with permission from Ref. [99], copyright 2019, Taylor & Francis. (**3**) SEM images of PVDF NFs with different concentrations (**a**) 29 wt%; (**b**) 32 wt%; (**c**) 35 wt%. Reprinted with permission from Ref. [102], copyright 2011, Polymer. (**4**) WAXD (**a**) and FTIR spectra of PVDF fibrous membranes (**b**). Reprinted with permission from Ref. [102], copyright 2011, Polymer. (**5**) XRD patterns of PVDF NFs at ambient temperature (**a**) 15 °C (**b**) 25 °C (**c**) 35 °C (**d**) 45 °C. Reprinted with permission from Ref. [103], copyright 2008, Walter De Gruyter GmbH. (**6**) FE-SEM images of PVDF NFs prepared at different ambient temperatures. Reprinted with permission from Ref. [103], copyright 2008, Walter De Gruyter GmbH.

These parameters are mainly determined by the properties of the polymer solution, usually including the molecular weight of PVDF, the concentration of PVDF solution, the solvent, the solution conductivity, and the surface tension. The molecular weight of PVDF affects the viscosity of the solution. When PVDF with a higher molecular weight is added to the solvent, a solution with higher viscosity is produced. A solution with high viscosity cannot smoothly pass through the needle tip and spin normally. In addition, an increase in viscosity gradually changes the bead shape from spherical to spindle until smooth fibers are obtained [97]. Magniez et al. [98] found that higher-molecular-weight PVDF can produce more β phase, and this result was also demonstrated in the study of Zaarour et al. [99]. When the molecular weight increases, the viscosity increases, leading to a longer spinning time, the evaporation time of the solvent becomes longer, and the stretching time of the polymer solution increases, so the β-phase content increases. Therefore, in order to obtain a large amount of β phase, the molecular weight can be increased, but pinholes can be blocked due to increased viscosity, so it should not be too high. Although increasing the diameter of the needle prevents clogging, the fiber diameter increases. Surface morphology and piezoelectric properties are shown in Figure 5(1,2).

The concentration of PVDF is generally in the range of 10–25%, and different concentrations of PVDF solutions produce different fiber morphologies [100]. Lower concentrations of PVDF solution produce polymer particles or nanoparticles when passing high-voltage electricity. This technology is the electrospray method rather than electrospinning, and when the concentration increases to a certain value, continuous NFs are produced. This is electrospinning technology, which produces smooth and continuous NFs in the optimal concentration range [101]. Similarly, the concentration of PVDF also affects the viscosity, and the stretching effect of the electric field on the polymer is more obvious when the viscosity increases. When the concentration reaches the best value, the formed fiber morphology is smooth and uniform and the content of β phase is the highest. When the concentration exceeds this value, the tensile effect is reduced due to the increase in viscosity, and the content of β phase is reduced. This can be proven from the study of Zhong et al. [102]. Fiber surface morphology and β-phase content are shown in Figure 5(3,4).

When using polar solvents to dissolve PVDF such as dimethylacetamide (DMAc), DMF, etc. [104], in the case of the same temperature and pressure, the saturated vapor pressure of these solvents is relatively low, so ACE with higher saturated vapor pressure is also selected as a solvent to dissolve PVDF for electrospinning. In this way, due to the higher vapor pressure of the solution, the complete evaporation of the solvent in the process of electrospinning can obtain smoother fibers and reduce the formation of beaded structure, which is conducive to the formation of β phase. Generally, the mixed solution of DMF and ACE with a ratio of 6:4 is used as the solvent [100]. 

The conductivity of the solution mainly depends on the polymer type, the solvent, and the added electrolyte. The conductivity of a polymer solution can be changed by adding a small amount of salt or electrolyte to the solution. When the charge density of the jet increases as the conductivity increases, the jet is stretched more fully, resulting in a smoother fiber [101]. Surface tension is an important factor affecting fiber properties: reducing surface tension can produce smoother fibers, and adding surfactants to the solution can reduce surface tension.

#### 2.2.3. Environmental Parameters

Environmental parameters can be divided into two parameters: ambient temperature and relative humidity. An increase in ambient temperature leads to faster solvent evaporation, which reduces the surface tension and viscosity of the solution. Zaarour et al. [105] found that with an increase in temperature, the diameter of the fiber becomes smaller and the surface becomes rough. This is because DMF with low vapor pressure hinders the evaporation of ACE with high vapor pressure when a DMF/ACE mixed solution is used as a solvent. The content of β phase from 20 °C to 60 °C was studied, and it was found that the content of β phase was the highest at 20 °C, and the content of β phase decreased with the increase in temperature. However, in the study of Huang et al. [103], it was found that the content of β phase was the highest at 25 °C. The increase in temperature led to the decrease in surface tension and viscosity, thus affecting the stretching of the solution, which was the main reason for the highest content of β phase being at 25 °C. Surface topography and XRD spectra are shown in Figure 5(5,6). 

Relative humidity also affects the evaporation of solvent. When the humidity increases, the evaporation of solvent is incomplete, which affects the surface morphology of fibers. Some studies have found that when the relative humidity increases from 2% to 62%, the content of β phase increases from 55% to 73.06% [106]. Higher relative humidity leads to increased water on the surface of the jet, which prolongs the stretching time, and the nucleation and growth time of the β phase is long, which increases the content of the β phase.

### 2.3. Effect of Adding Functional Particles on Piezoelectric Properties of PVDF NFs

The piezoelectric properties of PVDF NFs can be improved by adding various additives. Various nanomaterials can be easily added into PVDF solutions by electrospinning. The various additives can be divided into inorganic piezoelectric particles, metal–organic framework (MOF), conductive particles, organic additives, etc. Table 2 lists the influence factors of some additives, the content, the average diameter of the obtained fiber membranes, the peak voltage, the elastic modulus, and their related applications.

#### 2.3.1. Inorganic Piezoelectric Material

**Figure 6:**
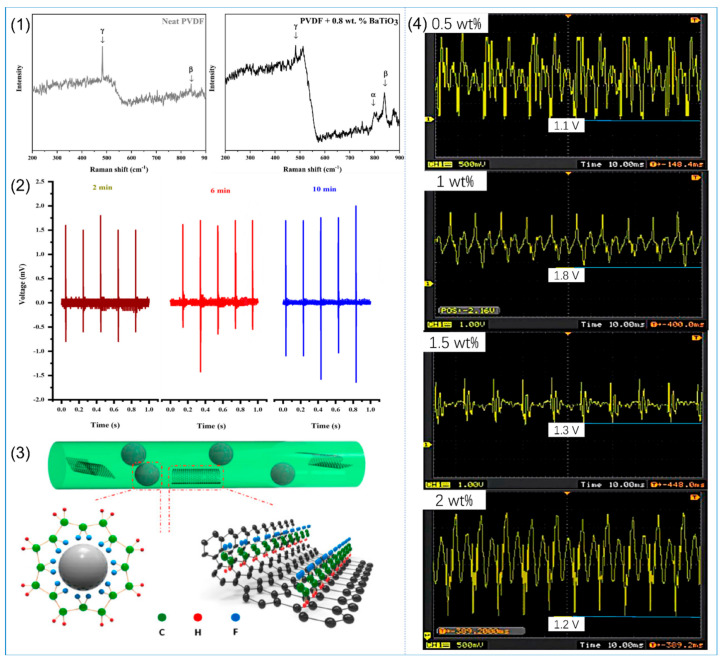
(**1**) Raman spectroscopy results of pure PVDF and PVDF containing 0.8 wt% BaTiO_3_. Reprinted with permission from Ref. [117], copyright 2021, Elsevier. (**2**) Voltage curve of PVDF−BatiO_3_−Ag composite as a function of time. Reprinted with permission from Ref. [117], copyright 2021, Elsevier. (**3**) Schematic diagram of modified nanographene to promote β−phase generation. Reprinted with permission from Ref. [111], copyright 2021, MDPI. (**4**) the piezoelectric signals of the fiber membrane when different amounts of modified nanographene were added. Reprinted with permission from Ref. [111], copyright 2021, MDPI.

As the most typical inorganic piezoelectric material, PZT is very suitable as an additive to improve the piezoelectric properties of PVDF. Adding 20 wt% PZT to PVDF for electrospinning can obtain a fiber film with the best piezoelectric properties [107]. However, because lead-based piezoelectric materials are harmful to human health and are not conducive to recovery and carry the possibility of harm to the environment, research on lead-free piezoelectric materials is now a hot spot. A study on PVDF NFs with different concentrations of BaTiO_3_ found that the piezoelectric output voltage of PVDF NFs containing 16 wt% BaTiO_3_ was 1.7 times that of pure PVDF under the same test environment [108]. A similar conclusion can also be obtained in the study of Dashtizad et al. [117]: the content of β phase in PVDF NFs with 0.8 wt% BaTiO_3_ is the highest (90%), as shown in Figure 6(1), and silver particles are added by ultraviolet irradiation of AgNO_3_ aqueous solution containing BaTiO_3_. By controlling the number of silver ions added and the irradiation time, this method can increase the content of β phase and the piezoelectric properties of PVDF, as shown in Figure 6(2). The results showed that the output voltage of PVDF NFs with ZnO nanoparticles was increased by 1.1 V compared with pure PVDF NFs [109]. This was also confirmed in the study of Li et al. [118], in which nanofiber membranes prepared by electrospinning technology after mixing PVDF with ZnO nanoparticles showed smaller fiber diameters and increased output voltage. In addition, modified ZnO can also improve the piezoelectric properties of PVDF NFs. Graphene has excellent optical, electrical, and mechanical properties; combining it with other materials can improve various physical and chemical properties [119,120]. Hasanzadeh et al. [110] synthesized graphene–zinc oxide nanocomposite (G–ZnO) by the hydrothermal method and added it to PVDF for electrospinning, and used XRD and TFIR to characterize the β phase of the NFs. It was found that the β-phase content of the composite was higher, and the piezoelectric output performance of this composite was better.

#### 2.3.2. Conductive Particles

The addition of conductive particles can also improve the piezoelectric properties of PVDF NFs. Because the addition of conductive particles increases the surface charge density of the jet during electrospinning, the bending whipping effect of the jet is enhanced, the fiber is better stretched, and the content of β phase is relatively improved. Li et al. [111] studied the piezoelectric properties of electrospun composite NFs with the addition of AgNO_3_, FeCl_3_·6H_2_O, and modified graphene to PVDF. The relevant data are shown in Table 2. The results showed that the piezoelectric signal of the composite fiber membrane with 0.3 wt% AgNO_3_ was the highest when the number of silver ions was low, and the piezoelectric signal of the composite fiber membrane with 0.3 wt% AgNO_3_ was the highest when the number of silver ions was low. As the solution conductivity increases, its level jet whip dynamic instability is improved, and because its content is more than 0.3 wt%, the fluid resistance due to the increase in surface tension increases, and as a result of the Ag^+^ and NO^3-^ gathered, results in an uneven surface charge releasing; additionally, dynamic instability increases, causing a whip against solution stretching. The content of β phase decreases instead, so high AgNO_3_ concentrations are not conducive to the improvement of piezoelectric properties of PVDF composite NFs. FeCl_3_·6H_2_O was also added to PVDF and its piezoelectric properties were studied. With the increase in FeCl_3_·6H_2_O content, the piezoelectric output voltage of the fiber membrane reached its highest (4.8 V) when the content reached 0.8 wt%. Moreover, this addition also affects the β nucleation of PVDF, since the specific interactions near the Fe/PVDF interface can induce PVDF β nucleation [121], and the strong hydrogen bond interaction formed between the water molecules in FeCl_3_·6H_2_O and polar CF_2_ may be the driving factor for β nucleation. After adding modified graphene, due to the van der Waals force between carbon atoms, they are easy to collect. In order to make it more evenly dispersed into the PVDF solution, rare earth element La is added to form La−C bonds to make it stable, and the diameter of PVDF fibers after adding modified graphene is smaller, and the conductivity is improved. Among them, in the PVDF with the addition of 1 wt% modified graphene, β-phase content was the highest and the piezoelectric output voltage was the highest, as shown in Figure 6(4). In addition to promoting jet stretching by increasing solution conductivity, adding nanographene can also promote the formation of β phase; the surface of nanographene in the interface interaction forms β phase of PVDF, as shown in Figure 6(3). It was also found that the addition of unmodified nanographene affected the movement of PVDF chains due to the aggregation effect, resulting in a lower β-phase content than pure PVDF. Adding carbon nanotubes (CNTs) can also improve the sensor performance of fibrous PVDF membranes, and piezoelectric properties of the PVDF with CNTs added for electrostatic spinning used a sound-absorbing device. The results found that the specific surface area of a fiber membrane improved, increasing the acoustic contact and improving the crystallinity of β phase; making it in the low frequency area of the sound waves can also result in good absorption [112]. In another study, CNTs were added to PVDF/potassium sodium niobate (KNN). The results showed that the addition of 0.1% CNTs could significantly improve the piezoelectric performance, with an output voltage of 23.24 V, a current of 9 μA, and a power density of 52.29 μW/cm^2^. Since the addition of CNTs improves the electrical conductivity of the solution, the jet stretches better, resulting in more β phase [122]; this can also be confirmed in the work of Hehata et al. [123]. Boron nitride nanosheets (BNNs) can also be added to PVDF to improve the piezoelectric properties of PVDF [113]. The results show that the piezoelectric properties of PVDF/BNNs composite nanofiber film are improved, and the triboelectric effect between the metal electrode and the composite fiber film also helps to improve the electrical output.

#### 2.3.3. Other Additives

**Figure 7:**
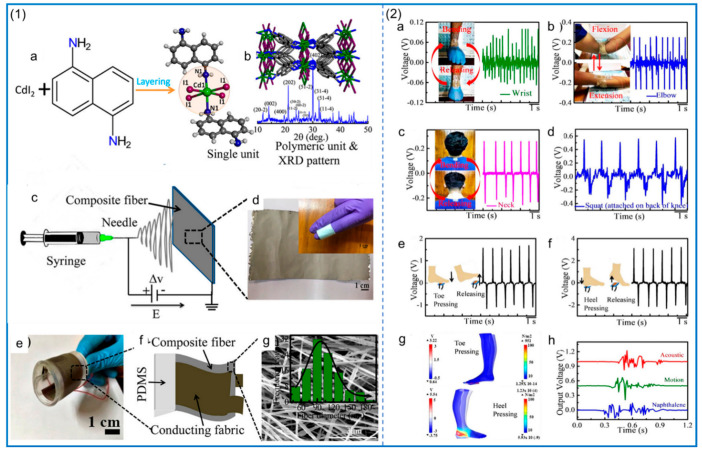
(**1**) The macroscopic and microscopic structure and state diagram of the material, (**a**) the schematic diagram of the synthesis route of two-dimensional MOF (CdI_2_−NAP) and its unit structure. (**b**) MOF structure (top), XRD (bottom). (**c**,**d**) electrospinning. (**e**,**f**) photos of C−PNG. (**g**) FE-SEM images of composite NFs, inset shows a histogram of fiber diameter distribution. Reprinted with permission from Ref. [126], copyright 2021, American Chemical Society. (**2**) Analysis of piezoelectric properties during human movement, (**a**) voltage responses of C−PNGs attached to a wrist, (**b**) elbow, (**c**) neck, (**d**) knee, (**e**) toe, and (**f**) heel movements, the arrows represent pressure and release. (**g**) Simulation based on FEM. (**h**) Voltage response curves of C−PNGs attached to the throat during “acoustic,” “motor,” and “naphthene” vocations. Reprinted with permission from Ref. [126], copyright 2021, American Chemical Society.

In addition, MOF particles can be added to enhance the piezoelectric properties of PVDF. Generally speaking, we hope to obtain composite fiber membranes with better performance by increasing the loading of MOF. However, when the MOF content is too high, it makes electrospinning difficult to carry out, so the performance of the fiber membrane decreases [124]. Moghadam et al. [114] found that the NFs produced by adding zirconium-based MOF to PVDF can greatly improve its piezoelectric properties. The β-phase content of PVDF with Zr-based MOF increased by 16%, and the output voltage of PVDF with 5 wt% MOF was found to be the highest when it was used for human arterial pulse detection. In addition, Zr-based MOF can also be used for SO_2_ detection, and the results show that it has high sensitivity and excellent flexibility [125]. Roy et al. [126] incorporated 2D MOF into PVDF, and the structure of MOF is shown in Figure 7(1). The composite fiber-based piezoelectric nanogenerator (C-PNG) was prepared by the electrospinning method. The coupling effect between the MOF and PVDF NFs led to the enhancement of the piezoelectric performance of the composite. The output voltage can reach 6 V—the corresponding motion test results are shown in Figure 7(2)—which gives the material good applicability in the field of low-frequency noise detection. The addition of organic additives to the PVDF solution can also enhance the electroactive phase of PVDF. Xue et al. [116] studied the effect of dioctadecyl dimethyl ammonium chloride (DDAC) on the piezoelectric properties of PVDF, and the results showed that the β-phase content of PVDF composite NFs with 4% DDAC was 39.1% higher than that of PVDF NFs without DDAC.

## 3. Preparation of PVDF Copolymer NFs by Electrospinning

**Figure 8:**
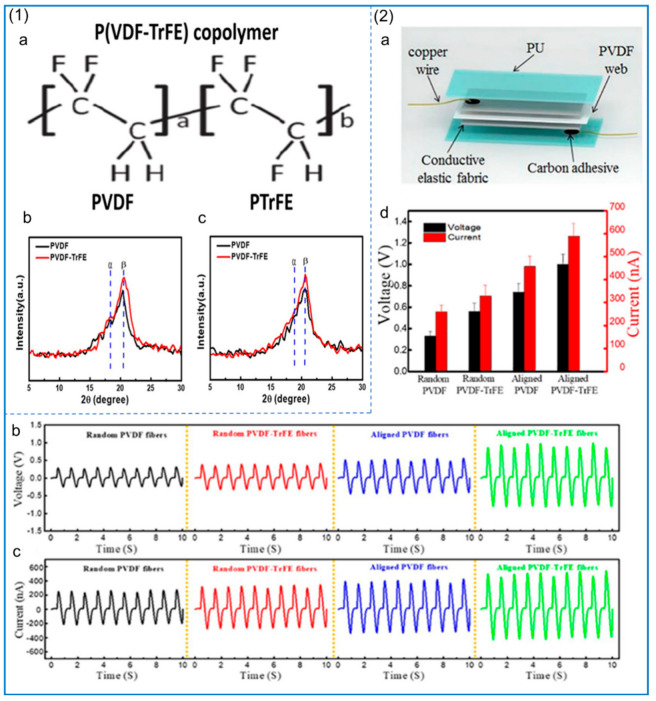
(**1**) Analysis of PVDF and PVDF–TRFE NFs, and (**a**) Molecular structure of PVDF–TRFE. (**b**) XRD patterns of randomly oriented PVDF and PVDF–TRFE NFs. (**c**) XRD patterns of PVDF and PVDF–TRFE fibers arranged in order. Reprinted with permission from Ref. [127], copyright 2020, SAGE Publications Ltd. STM. (**2**) PNG analysis, (**a**) Schematic diagram of the structure of PNG. Statistical results of (**b**) output voltage, (**c**) output current, (**d**) average voltage, and current generated by PNG during folding and release at 90° bending. Reprinted with permission from Ref. [127], copyright 2020, SAGE Publications Ltd. STM.

The copolymers of PVDF are mainly poly (vinylidene fluoride–trifluoride) (PVDF–TRFE), poly (vinylidene fluoride–tetrafluoride) (PVDF–TFE), and poly (vinylidene fluoride–hexafluoride) (PVDF–HFP). The piezoelectric properties of these polymers, as well as their biocompatibility and flexibility, are very excellent. Zhang et al. [127] made two kinds of piezoelectric nanogenerators (PNG) based on PVDF and PVDF–TRFE, and studied the mechanical and piezoelectric properties of PVDF and PVDF–TRFE NFs as well as the crystallinity of β phase. The structure and XRD analysis are shown in Figure 8(1), and the piezoelectric performance test of the piezoelectric nanogenerator is shown in Figure 8(2). The results show that the mechanical properties and piezoelectric properties of PVDF–TRFE are better than those of pure PVDF. For the piezoelectric nanogenerator made of PVDF–TRFE, its β-phase content is higher and the output voltage is higher. In addition, compared with the performance of electrospinning PVDF and PVDF–TRFE with polyethylene oxide (PEO) and LiCl, the β-phase content of PVDF–TRFE with PEO is higher than that of PVDF with the same addition [128], and the addition of LiCl improves the conductivity of the solution and also leads to an increase in the β-phase content. The fabricated PVDF–TRFE/PEO composite nanofiber membrane can achieve an instantaneous power of 40.7 μW/cm^2^ under a mechanical force of 1.58 N.

**Figure 9:**
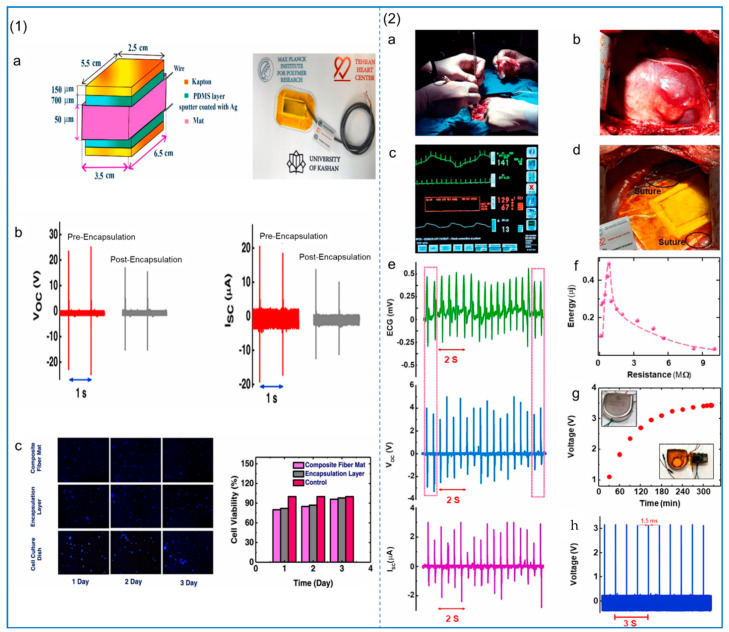
(**1**) PNG structure diagram and its voltage, current, and biocompatibility test, (**a**) Schematic diagram and photo of PNG. (**b**) Open−circuit voltage and short−circuit current before and after PNG packaging. (**c**) Fluorescence images and viability of MEF cells after 1, 2, and 3 days of culture on composite fibrous membranes, encapsulated layers of PNG, and cell culture dishes. Reprinted with permission from Ref. [129], copyright 2021, Elsevier. (**2**) Self−powered cardiac pacemakers and (**a**) surgical procedures in animal experiments. (**b**) Photographs of the heart and (**c**) ECG signals and blood pressure recorded simultaneously. (**d**) PNG stitched to the epicardium facing the left outdoor lateral wall (**e**) In vivo ECG signal, open−circuit voltage, and short-circuit current of PNG. (**f**) Energy generated by PNG. (**g**) Charging curve of a 100 μF capacitor charged by PNG. (**h**) Pacing pulses generated by a PNG−powered pacemaker. Reprinted with permission from Ref. [129], copyright 2021, Elsevier.

The addition of other particles can also improve the piezoelectric properties of PVDF–TRFE. ZnO and reduced graphene oxide (rGO) were added to PVDF–TRFE using electrospinning technology to prepare NFs and fabricate PNG for self-powered cardiac pacemakers [129]. The mass ratio of ZnO to rGO is 9:1. The PNG structure and its voltage, current, and biocompatibility test are shown in Figure 9(1). The results show that the PVDF nanofiber membrane containing 0.1 wt% ZnO/rGO reaches the maximum output power of 138 ± 2.82 μW/cm^2^. An electrical energy of 0.487 μJ can be collected from each heartbeat, as shown in Figure 9(2), for surgical pictures and related tests, which is a high collection rate. The high-speed roller speed during the electrospinning of PVDF–HFP can produce nanofiber films with smaller fiber diameters and more β-phase content [130]. A similar conclusion can be obtained in the study of Conte et al. [131], where the piezoelectric properties of PVDF–HFP fiber membranes can be enhanced by optimizing processing conditions such as tensile rate and temperature. Wu et al. [132], by incorporating barium calcium zirconia titanate (BCZT) into PVDF–HFP and using electrospinning technology to fabricate NFs, significantly improved its piezoelectric properties, and the maximum power density reached 161.7 Mw/cm^2^. The PVDF–HFP/ZnO NF piezoelectric sensor fabricated by the addition of ZnO has excellent performance and an ultra-high sensitivity of 1.9 V/kPa, which is due to the combination effect between the electric dipole inside the fiber and the asymmetric lattice of ZnO to improve the piezoelectric performance [133]. Pinnamma et al. [134] fabricated ZnO and TiO_2_ containing PVDF/PVDF–HFP core–shell-structured NFs based on electrospinning, which produced output voltages of up to 14 V and had a dielectric constant that was five times higher than that of pure polymers.

## 4. Preparation of Other Piezoelectric NFs by Electrospinning

**Figure 10:**
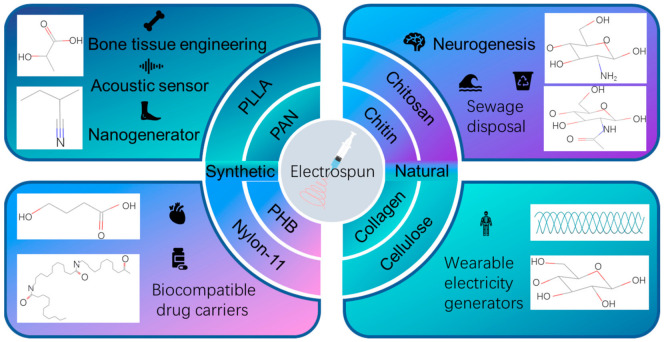
Structures and applications of other piezoelectric NFs prepared by electrospinning.

In addition to PVDF, there are many other electrospun piezoelectric fiber membranes, such as poly-l-lactic acid (PLLA), which is a biodegradable polymer derived from plant alkalis, poly (3-hydroxybutyric acid) (PHB), which is also a biodegradable polymer, PAN, Nylon-11, collagen, cellulose, chitin, chitosan derived from chitin, etc. A subset of spinnable piezoelectric polymers and their applications are listed in Figure 10.

### 4.1. Preparation of Synthetic Piezoelectric Polymers by Electrospinning

PLLA has good biocompatibility, which can be proved by the FDA’s approval of PLLA injection for human tissue in 2004, and has a good prospect in the application of some implantable devices. Compared to PVDF, the polarization direction of PLLA in the piezoelectric domain is not the same, and the polarization direction is parallel to the plane where the shear stress is applied, leading to the shear piezoelectric property of the polymer. This is different from PVDF fiber membranes that lack a shear strain response, and PLLA can be more effectively applied in shear piezoelectrics [135]. PLLA NFs prepared by electrospinning have different effects on their piezoelectric properties under different surface morphologies and different heat treatments [136]. Fiber diameter and heat treatment have a great impact on the transverse and longitudinal output voltage of PLLA NFs, which affects the differentiation of stem cells. Orthogonal and shear piezoelectric properties affect neurogenesis and osteogenesis, respectively, and the improvement of piezoelectric properties enhances the regeneration process accordingly. Zhao et al. [137] also prepared PLLA NFs that can reach a current of 8 pA and a voltage of 20 mV, and prepared a blood pulse sensor, which can produce an output current of 2 pA and can effectively detect blood pulses. The addition of hydroxyapatite (HAP) to PLLA can also enhance the related ability of PLLA in cell cultures, where electrospinning is performed to prepare NFs [138]. These fiber matrices have good performance as osteocyte culture substrates. The results showed that pure PLLA NFs did not release substances that were toxic to cells. Addition of 0.5% (w/v) HAP particles to PLLA fibrous membrane scaffolds not only promoted the attachment and proliferation of precellular Mc3t3-e1 mouse osteoblasts, but also increased the expression of osteocalcin mRNA and the degree of mineralization of cells after 14 and 21 days of culture on the scaffolds, respectively. Therefore, PLLA/HAP fibrous membranes may be the preferred material for bone tissue engineering.

PAN is an amorphous vinyl polymer that contains a cyano (–CN) group in each repeat unit. It has a zigzag and helical structure and has broad applications in many fields such as the environment, energy, and drug delivery [139,140,141,142,143]. The zigzag has an all-trans structure (TTT) and a dipole moment of 3.5 Debye [144]. Although PAN has piezoelectric properties, the piezoelectric constant is much lower than other polymers such as PVDF [56]. Most studies have used PAN as a carrier for piezoelectric materials [145,146]. Methods to enhance the piezoelectric properties of PAN have been studied. PAN nanofiber films prepared by electrospinning have better piezoelectric properties than PVDF. A small piece of PAN NF can generate an output voltage of 2.0 V and an output current of 1.1 μA. The residual charge on PAN NFs also contributes to energy conversion [147]. This can also be confirmed in the study by Street et al. [58]. Among the PAN NFs with ZnO addition, the pressure-sensing ability and energy harvesting efficiency of ZnO/PAN composite NFs are 2.7 times higher than those of pure PAN NFs [146]. Using carbonized PAN/barium strontium titanate (BTO) NFs to make the sensor, the pressure-sensing sensitivity was increased by more than 2.4 times when BTO nanoparticles were added to the PAN nanofiber film due to the synergistic effect of piezoelectric and triboelectric effects [148]. Airagi et al. [149] added CuO to PAN to prepare an electrospun fiber nanogenerator, and found that the piezoelectric performance of the nanogenerator increased with an increase in CuO addition. The main reason for this is that CuO provides a conductive path for PAN, which makes the charge on the electrode increase and the energy collection efficiency increase. The PAN nanofiber film containing 0.5% CuO had an output voltage of 5 V and an output current of 0.172 μA, and the power density of the nanogenerator was 0.215 μW/cm^2^. In situ polarization and stretching via an electrospinning process causes the –CN groups of PAN to rearrange on both sides of the polymer chain, increasing the number of electroactive conformations. PAN NFs can also be used for acoustic detection. Poon et al. [150] studied the ability of PAN NFs in acoustic detection and the ability of electrospun PAN NFs to accurately detect low- and medium-frequency sounds (100–600 Hz) at a medium sound pressure level (60–95 dB), which covers the main sound spectrum in our daily activities. Compared with acoustic sensors made from PVDF NFs under the same conditions, the PAN device has a wider response bandwidth, greater sensitivity, and higher fidelity, which indicates that PAN plays an important role in acoustic detection of NFs.

PHB is a biodegradable material that has good biocompatibility. Based on the sustainable development strategy, it can be used to replace polypropylene and polyethylene. However, the piezoelectric coefficient of PHB is very low. Jiang et al. [151] prepared PHB NFs with multi-walled CNTs (MWCNTs) by the electrospinning method. Compared with pure PHB nanofiber scaffolds, PHB/MWCNTs composite nanofiber scaffolds contain higher β-shaped crystal content. The piezoelectric charge constant and induced potential measured on the surface of the nanofiber scaffold indicate that the addition of MWCNTs and thermal stretching treatment to the PHB nanofiber matrix helps to improve the piezoelectric properties of PHB. Chhernozem et al. [152] prepared PHB NFs with rGO. The surface potential distribution of PHB–rGO fibers was more uniform, and the average voltage increased from 33 ± 29 mV to 314 ± 31 mV compared with the pure PHB nanofiber membrane. Further increases in rGO content lead to increased α crystal deformation and prevents zigzag chain formation, which results in decreased crystallinity and pressure sensitivity of PHB scaffolds. The degradable electrospun composite scaffolds of polyaniline (PANI) and PHB have better piezoelectric performances. The doping of piezoelectric polymer PHB and conductive PANI can significantly improve the piezoelectric charge coefficient and surface potential of pure PHB scaffolds. Compared with pure PHB scaffolds, the surface potential under cyclic mechanical stress at a frequency of 4 Hz is increased by 4.2 times and 3.5 times, respectively [153]. Karpov et al. [154] also reported the piezoelectric response of PHB NFs and studied the piezoelectric properties of polycaprolactone (PCL), PHB, and PHB–PANI, and found that PCL had the worst piezoelectric properties and PHB–PANI had the best piezoelectric properties.

Nylon (polyamide) is a general term for thermoplastic resin containing repetitive unit amide group (-[NHCO]-) in the molecular backbone, among which Nylon-11 of odd nylon has better piezoelectric properties, and there are four crystalline phases of Nylon-11, namely α, α’, γ, δ’. Only the δ’ phase has better piezoelectric properties. This is because α and α’ phases of Nylon-11 do not have piezoelectric properties due to different grain orientation and also have strong hydrogen bonds that cannot be electrically polarized. The γ phase has a very weak polarity, so the piezoelectric properties are very poor [155]. The δ’ phase has randomly distributed hydrogen bonds, so dipoles are generated when electrically polarized. However, it is difficult to generate the piezoelectric δ’ phase by traditional production methods of Nylon-11 fibers. The cooling rate determines the crystal structure of Nylon-11, and it has been found that the faster the cooling, the higher the content of δ’ phase [156]. The high-vapor-pressure and low-boiling-point solvents trifluoroacetic acid (TFA) and ACE were used for electrospinning. The reason why ACE is added is that TFA forms hydrogen bonds with amide groups on the Nylon-11 chain, some of them are left in the Nylon-11 fibers when they evaporate, and the hydrogen bond between ACE and TFA is stronger. ACE removes TFA from the Nylon-11 as it evaporates. Anwar et al. [157] found that Nylon-11 NFs prepared by electrospinning with TFA and ACE as solvents could generate an output voltage of 6 V under the action of mechanical force.

### 4.2. Preparation of Natural Piezoelectric Polymers by Electrospinning

Although natural polymers are popular in applications associated with medical products [158,159,160,161,162], there are also some natural piezoelectric polymers in addition to the above-mentioned polymers, such as collagen, cellulose, chitin, chitin-derived chitosan, etc. These polymers are extracted from organisms in nature, so their biocompatibility is very good and their toxicity is very weak.

Collagen comprises the main amino acid glycine, proline, hydroxyproline, and alanine; these amino acids have piezoelectric properties [163]. Collagen is the main component of connective tissue, accounting for nearly a quarter of total protein in human body, three-fourths of the human body skin dry weight, more than 90% of human tendon and corneal tissue, and nearly 80% of organic matter in bone [164]. Twenty-nine collagen types have been identified, of which type I collagen in particular may be a good candidate for biomedical applications, such as scaffolds for wound dressings and tissue engineering. Rho et al. [165] prepared a collagen nanofiber matrix by the electrospinning method for wound dressing. This nanofiber has the characteristics of wide pore size distribution, high porosity, good mechanical strength, and a high surface area-to-volume ratio, which is conducive to cell attachment, growth, and proliferation. They found that NFs coated with type I collagen promoted adhesion and spreading of human keratinocytes.

Cellulose is a kind of polysaccharide. Constituting the basic unit of cellulose is glucose, which can be found in plants, but due to its low piezoelectric constant, cellulose will, generally, be broken after being mixed with piezoelectric materials during the pouring process in the film production of nanogenerators, for example, the combination of cellulose and MoS_2_ can greatly improve mechanical strength and piezoelectric properties [65]. There are not many studies on the preparation of cellulose by electrospinning technology because cellulose has poor spinnability. Usually, cellulose derivatives such as cellulose acetate are used for electrospinning to prepare NFs, and then hydrolyzed to obtain cellulose NFs.

Chitin is a polysaccharide substance extracted from the shells of arthropods, the cell walls of fungi, and the wings of butterflies. Its structure is very similar to that of cellulose, which is a polymer of six-carbon sugar. The basic unit of chitin is acetylglucosamine. Chitin exists in three crystal forms, namely α, β, and γ, among which α has piezoelectric properties [166]. Treet et al. [167] demonstrated that chitin NFs prepared by electrospinning have higher crystallinity and piezoelectric properties. The crystallinity and piezoelectric properties of chitin can be adjusted by controlling the parameters of electrospinning, which is helpful for the adsorption of heavy metals [168]. This means chitin has good application prospects in sewage treatment due to its convenient access.

Chitosan is the product of N-deacetylation of chitin, and is similar to chitin and cellulose in structure. The monomer is glucosamine, which is the only basic polysaccharide in the group of natural polysaccharides due to its free amino group [169]. Chitosan has great potential for drug release. Dexamethasone (DEX) was loaded onto chitosan nanoparticles and mixed with a PCL solution, then ZnO nanorods were mixed with the PVDF solution; mixing two kinds of solution using a double-electrospinning method allowed for the preparation of compound electrospun NF membranes. A DEX controlled-release scaffold was fabricated to induce the osteogenic differentiation of bone tissue-engineered mouse bone marrow mesenchymal stem cells (MSCS); this method of controlled release of DEX was proven to be beneficial to osteogenic differentiation [170]. 

## 5. Application of Electrospun Piezoelectric NF Membranes

**Figure 11:**
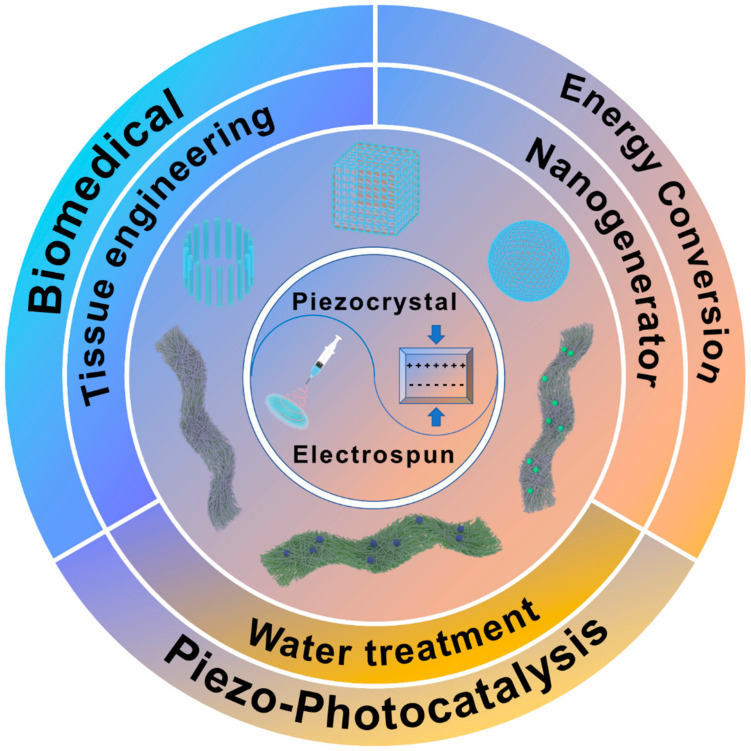
Application of electrospun piezoelectric NFs.

Because of their unique piezoelectric properties, good biocompatibility, and flexibility, piezoelectric fiber membranes in biomedicine, water treatment, piezoelectric photocatalysis, energy collection, nanogenerators, and air particle adsorption have very broad application prospects, as shown in Figure 11. In recent years, researchers have done a lot of related work in these areas [171,172,173,174]. 

### 5.1. Biomedical Applications

Compared with ceramic piezoelectric materials, many piezoelectric polymer materials have very good biocompatibility, and electrospun fibers have broad prospects in the fields of biomedicine and ergonomics. Since piezoelectric materials can generate electrical signals and electrical stimulation, they can be applied to tissue engineering scaffolds. Piezoelectric fiber membranes can also be used in biosensors, drug delivery platforms, and so on. Due to the self-electric function of piezoelectric materials, they can be used to replace implantable devices or wearable sensors that require battery replacement.

#### 5.1.1. Scaffold for Tissue Engineering

Tissue engineering scaffold materials refer to materials that can combine with living tissue cells and implant different tissues of organisms according to the function of specific replacement tissues. In order to enable seed cells to proliferate and differentiate, it is necessary to provide a cell scaffold composed of biological materials, which is equivalent to an artificial extracellular matrix. The preparation of tissue engineering scaffolds by electrospinning has great application prospects [175,176,177], for example, charge generation on the surface of PVDF–TRFE NF scaffolds induced neural stem cells to differentiate into multiple neuronal, oligodendrocytic, and astrocytic phenotypes simultaneously under underwater acoustic driving [178]. Compared to traditional biochemically mediated differentiation, the 3D neuron–glial interface induced by mechanical–electrical stimulation results in enhanced interactions between cellular components, leading to superior neural connectivity and function.

Electrospun scaffolds have great advantages over other scaffolds. These scaffolds generate a fiber network similar to the morphology of an extracellular matrix in vivo to support cell growth, proliferation and differentiation. PVDF and its derivative PVDF–TRFE NFs show relatively high piezoelectric constant values when synthesized by electrospinning [179,180]. Ins et al. [181] found that PVDF NF scaffolds can enhance neuronal cell alignment and neural synapses, which is due to the piezoelectric effect induced by cell contractility, which in turn affects cell behavior.

**Figure 12:**
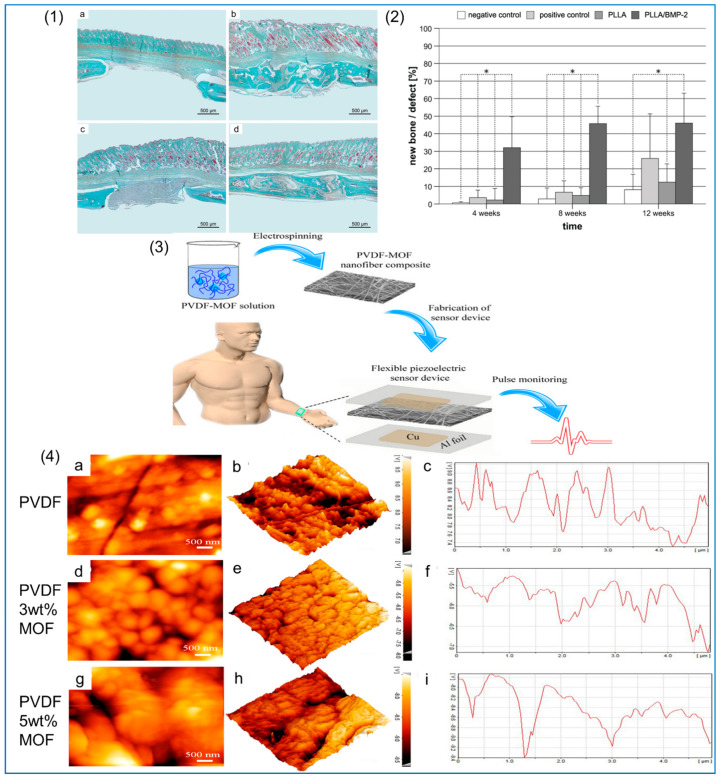
(**1**) Histological morphology at week 12: (**a**) negative control. (**b**) Positive control. (**c**) PLLA. (**d**) PLLA/rhBMP-2. Reprinted with permission from Ref. [185], copyright 2011, Public Library of Science. (**2**) The relationship between new bone formation and the whole defect area, * *p* < 0.05. Reprinted with permission from Ref. [185], copyright 2011, Public Library of Science. (**3**) Schematic diagram of arterial pulse-sensing device. Reprinted with permission from Ref. [114], copyright 2020, American Chemical Society. (**4**) Surface characteristics and piezoelectric response of PVDF NFs. (**a**,**d**,**g**) and (**b**,**e**,**h**) atomic force microscopy. (**c**,**f**,**i**) Voltage curves showing the piezoelectric response of the NFs. Reprinted with permission from Ref. [114], copyright 2020, American Chemical Society.

In addition to its use in neural tissue engineering, piezoelectric polymer materials also show promising applications in wound healing. Polyurethane/PVDF composite NFs were prepared by electrospinning for wound healing experiments in rats. The results showed that electrospinning technology had transformed the α phase of PVDF into β phase, and under the piezoelectric effect caused by mechanical deformation, the activity of fibroblasts in vitro and in vivo was improved [182]. In bone tissue engineering, Amaraju et al. [183] found that MSCS showed increased chondrogenesis and lower piezoelectric properties when cultured on electrospun PVDF–TRFE scaffolds compared to cells cultured on heat-treated PVDF–TRFE scaffolds. Similarly, PLLA and collagen are considered suitable piezoelectric materials for bone regeneration: their shear piezoelectricity is particularly relevant to the structure of collagen, the major organic component in bone. The use of PLLA as a bone substitute is advantageous, and PLLA implants can promote bone growth through piezoelectric effects [184]. PLLA NFs can stimulate cell differentiation and cell migration. A study by Schofer et al. [185] demonstrated that PLLA nanofiber scaffolds can promote cell migration and, thus, can achieve high cell density. However, they lack sufficient osteogenic stimulation to allow further differentiation of these cells, a problem that can be overcome by the incorporation of recombinant human bone morphogenetic protein-2 (rhBMP-2) into PLLA NFs. A portion of mouse skull resection was divided into four groups (group I: unfilled as negative control, group II: implanted with bovine cavernosum as positive control, group III: implanted with PLLA nanofiber scaffolds, and group IV: implanted with PLLA/rhBMP-2 nanofiber scaffolds). Morphometric measurements after 12 weeks are shown in Figure 12(1). After implantation of PLLA/rhBMP-2 nanofiber scaffolds, approximately 30% of the defect sites were filled with hard calluses after 4 weeks, which was significantly higher than what was observed in all other treatments. During the experiment, callus formation increased to 45% in the PLLA/rhBMP-2 group after 12 weeks, which was significantly higher than that in the negative control and PLLA groups, but there was no significant difference between the PLLA/rhBMP-2 group and the bovine spongiosum group after 12 weeks, although the average relative bone formation difference was about 20%, as shown in Figure 12(2).

Electrospinning techniques that combine mechanical stretching with spontaneous electrical polarization have generated unprecedented research enthusiasm in the preparation of fibrous polymers with piezoelectric properties. However, due to some disadvantages of piezoelectric polymers as tissue scaffolds and possible rejection in transplantation, systematic models for clinical application are still lacking.

#### 5.1.2. Biosensor and Drug Release Carrier

The polymer composite NFs prepared by the electrospinning method have micron-sized piezoelectric fiber meshes and have a good piezoelectric response. Due to the tensile and self-polarization of the fibers, the NFs produced by this process have good porosity and enhanced ferroelectric and piezoelectric properties. This has promising applications in self-powered implantable devices and electric clothing, especially small biosensors, which have very sensitive mechanical signal detection capabilities [186]. There is a lot of research on small self-powered devices for medicine. Moghadam et al. [114] developed a novel arterial pulse-sensing device using a microporous zirconium-based MOF embedded in PVDF NFs, as shown in Figure 12(3). By varying the content of MOF particles, the β crystal phase of PVDF is changed, and the piezoelectric performance of the sensor is controlled. In this device, the β-phase content of PVDF reaches up to 75%, the output voltage can reach 568 ± 76 mV, and the piezoelectric conversion sensitivity can reach 118 mV/N; its piezoelectric response and surface properties are shown in Figure 12(4). PVDF NFs can also be used in wearable flexible sensors that can monitor movement signals in the human body. Mahanty et al. [187] used the electrospinning method to prepare composite NFs by mixing MWCNTs and PVDF, and fabricated a skin sensor. The electronic skin sensor was installed on different parts of the human body, such as the wrist and arm muscles, and could generate a voltage of 5 V to monitor the physiological signals of the human body. The device can not only be used as an electronic skin pressure sensor, but can also be used in self-powered portable electronics.

**Figure 13:**
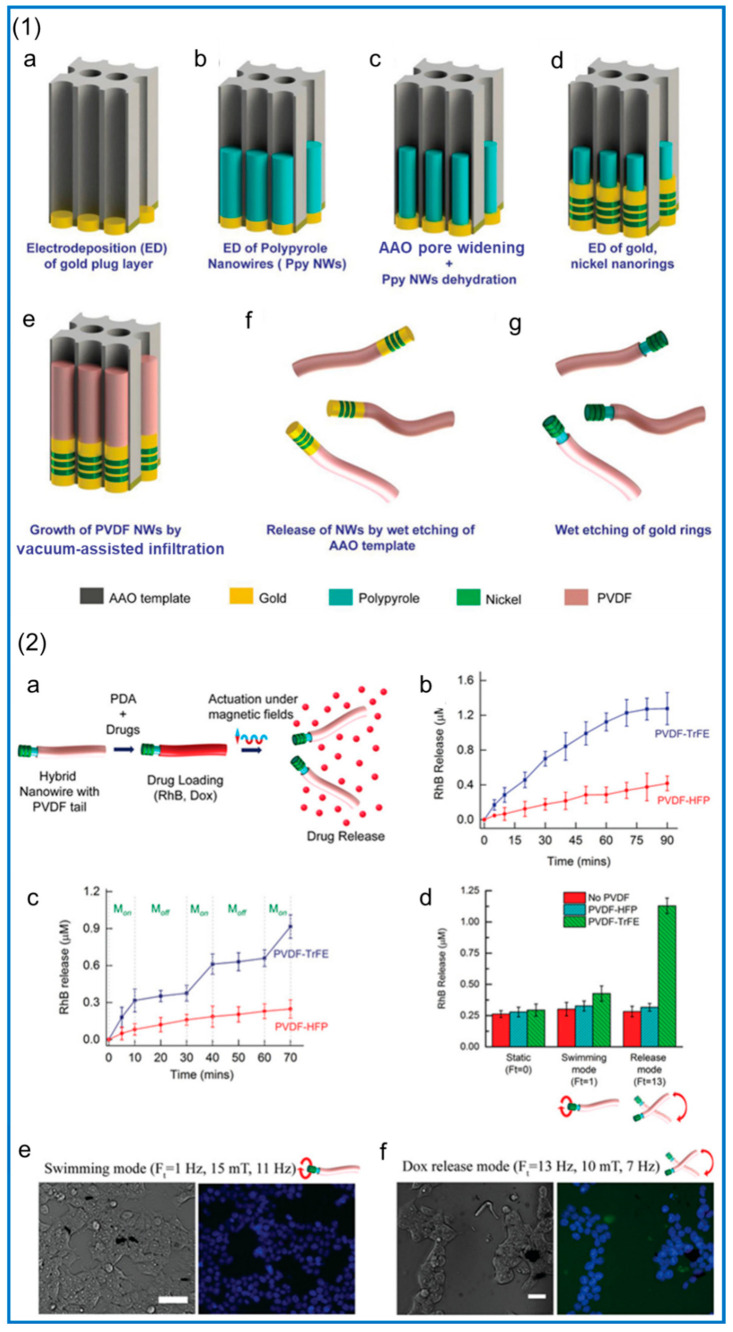
(**1**) Fabrication process of nano-electric eel, (**a**) Au sputtering and electrodeposition of Au plug layer in an AAO membrane, (**b**) electrodeposition of Ppy NWs, followed by (**c**) their dehydration and the pore widening of AAO template to allow for (**d**) sequential electrodeposition of Ni and Au nanoring segments. (**e**) Vacuum infiltration to form PVDF NWs, and RIE etching. Wet etching of (**f**) AAO template and (**g**) etching of gold nanoring segments to obtain released hybrid nano-electric eel. Reprinted with permission from Ref. [190], copyright 2019, John Wiley & Sons, Ltd. (**2**) Controlled drug release of mixed nano-electric eel. (**a**) Schematic representation of the magnetic drug release from mixed nano-electric eel. (**b**) Continuous release of RhB at 10 mT and 7 Hz. (**c**) The amount of RhB released with and without magnetic field. (**d**) Release plots of RhB in NWs without PVDF, with PVDF–HFP, and with PVDF–TRFE, bright-field and fluorescence images of cancer cells cultured with Adriamycin-coated PVDF–TRFE NWs in (**e**) swimming mode and (**f**) drug release mode. Reprinted with permission from Ref. [190], copyright 2019, John Wiley & Sons, Ltd.

The treatment of chronic diseases often requires a systemic drug release. Through the sustained release of drug concentration in the body for a long time, maintaining a certain level, we can accelerate the local regeneration or achieve the goal of continuous treatment for localized disease. This is a big challenge for conventional drug release systems [188]. Recent studies have found that the preparation of drug carriers by electrospinning is an excellent solution [189]. Piezoelectric materials can act as a switch for drug release. Due to its unique piezoelectric properties, the potential difference can affect the release of controlled drugs by generating opposite charges on opposite surfaces through the piezoelectric effect. Muhtaq et al. [190] designed a nanorobot as a drug delivery vehicle. The vehicle was a soft composite nanorobot that mimicked an electric eel, resembled a knifefish with a slender cylindrical body, and generated electricity as it moves. First, a gold coating is electrodeposited inside the anodized aluminum oxide (AAO) template, as shown in Figure 13(1a), to avoid electrolyte leakage during subsequent fabrication steps. Then, polypyrrole (PPy) NWs are electrodeposited, as shown in Figure 13(1b). Subsequently, electrodeposited PPy NWs contract radially in AAO pores, and then the template is reamed using a diluted NaOH solution. These two steps introduce a void between the PPy NWs and AAO pore walls, as shown in Figure 13(1c), and electrodeposit Ni/Au nanofragments around the PPy NWs, as shown in Figure 13(1d). Next, a solution containing the PVDF-based copolymer is filled into the template pores by vacuum permeation, and the template is subsequently dried and annealed to form NWs, shown in Figure 13(1e). Finally, the AAO template is wet etched to release hybrid nanoparticles, as shown in Figure 13(1f), and then the gold nanoring segment is etched to obtain the monomer hybrid nano-electric eel, as shown in Figure 13(1g). Incorporation of doxorubicin and rhodamine B (RhB) into the nanorobot’s tail portion by adsorption allows on-demand release from the tail surface under optimally tuned magnetic fields (10 mT and 7 Hz). Its drug release and related tests are shown in Figure 13(2e), we can see multiple nuclei (blue) without the presence of the drug Adriamycin, while in f, we can clearly see nuclei (blue) and released Adriamycin (green) in multiple locations.

### 5.2. Piezo-Photocatalysis

**Figure 14:**
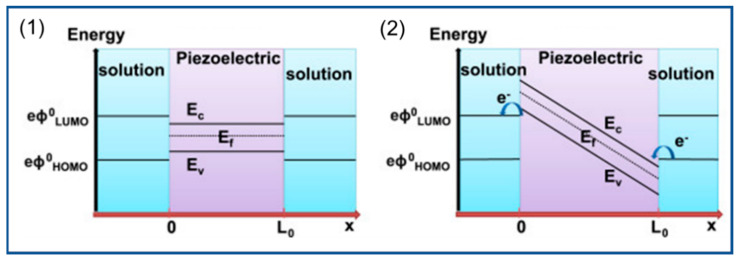
Band-structure changes before (**1**) and after (**2**) strain, and the energy shift on the occupied and unoccupied states. Reprinted with permission from Ref. [191], copyright 2015, Elsevier.

Due to the existence of dipole moments in piezoelectric materials, mechanical force causes the two opposite surfaces of the material to produce opposite charges to form a built-in electric field and generate a potential difference. This potential change changes the electronic energy level and drives the electrochemical reaction. For example, in photocatalytic water treatment, if the piezoelectric potential generated by the piezoelectric material is greater than the potential required for the REDOX reaction, the conduction band (CB) of the piezoelectric material is reduced below the highest-occupied molecular orbital (HOMO) of the solution in contact with it, and the oxidation reaction occurs when electrons are transferred from the HOMO to the CB. However, the valence band (VB) moves above the lowest-unoccupied orbital (LUMO), and the reduction reaction occurs when electrons are transferred from the VB to the LUMO, as shown in Figure 14 [191]. The preparation of piezoelectric photocatalyst nanofiber membranes by the electrospinning method not only improves catalytic activity but also solves the shortcomings of the catalyst that are not easy to recover.

**Figure 15:**
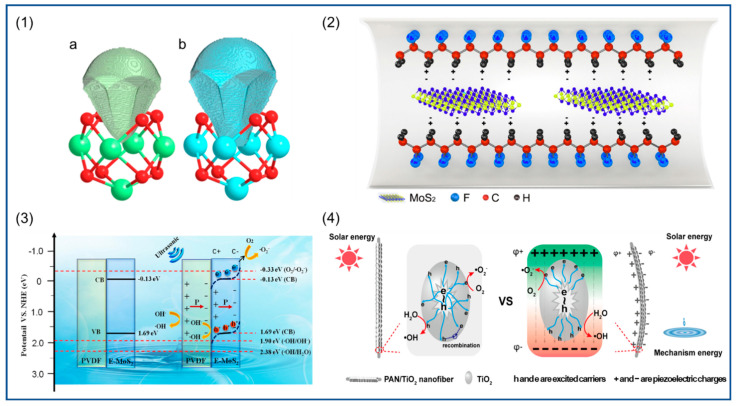
ELF basin analysis of the optimized geometry of (**1**) (**a**) Zr−oxo and (**b**) HF−oxo clusters. Reprinted with permission from Ref. [14], copyright 2021, John Wiley & Sons, Ltd. (**2**) Schematic diagram of β-phase formation in MoS_2_/PVDF. Reprinted with permission from Ref. [192], copyright 2021, Elsevier. (**3**) the mechanism of interfacial charge transfer process under the influence of band structure and band bending. Reprinted with permission from Ref. [192], copyright 2021, Elsevier. (**4**) Piezoelectric photocatalysis principle of TiO_2_/PVDF. Reprinted with permission from Ref. [196], copyright 2022, Elsevier.

Combining piezoelectric materials with photocatalysts with piezoelectric properties can result in composites that outperform both. Ma et al. [192] used electrospinning technology to prepare composite NFs of molybdenum disulfide (MoS_2_) nanosheets and PVDF, and their piezoelectric catalytic activity was confirmed by degradation of the antibiotic oxytetracycline. The electrostatic interaction between MoS_2_ nanosheets and –CH_2_ groups of PVDF promoted the transition from α phase to β phase. A schematic diagram of β-phase formation in MoS_2_/PVDF is shown in Figure 15(2), that is, MoS_2_ nanosheets induce β nucleation of PVDF and increase the piezoelectric potential, and Figure 15(3) illustrates the basic principle of their piezoelectric photocatalysis. In addition, MoS_2_ nanosheets are exposed to the surface of the NFs, which act as additional active sites to enhance the piezoelectric catalytic activity. In the study of enhancing photocatalytic activity, the use of piezoelectric polymer nanofibers as composite reinforcers is an effective strategy. Because most of the light catalysts produce electrons and holes before migrating to the catalyst surface recombination, the carrier recombination rate is higher, its photocatalytic performance is affected, and the built-in electric field generated by the piezoelectric materials can effectively prevent the electrons and holes in the compound to improve the catalytic performance. Titanium dioxide (TiO_2_) is a simple and stable photocatalyst. The catalytic performance of the composite fiber membrane is enhanced by loading TiO_2_ onto PVDF to prepare bilayer hollow NFs [193]. In another study, TiO_2_ was loaded on the surface of PbTiO_3_, a single-crystal fiber was prepared by the secondary hydrothermal method, and a PN junction was formed at the TiO_2_/PbTiO_3_ interface. Due to the piezoelectric properties of PbTiO_3_, under the action of ultrasonic waves, a built-in electric field was generated in the crystal, which promoted the carrier separation and improved the catalytic efficiency of TiO_2_. The catalytic efficiency of the composite fiber for methylene orange was increased by 60% compared with that of TiO_2_ [194]. Urairaj et al. [195] prepared TiO_2_-loaded PVDF NFs and, similarly, found that the photocatalytic efficiency for degradation of organic pollutants was greatly improved. In the study of Ding et al. [196], TiO_2_ loading onto PAN also resulted in the enhancement of photocatalytic efficiency, and its piezoelectric photocatalytic principle is shown in Figure 15(4). Piezoelectric catalytic filters are very useful in wastewater treatment, and can easily decompose wastewater and do not produce secondary pollutants in the treated water [197]. MoS_2_ was loaded on carbon fiber, and the composite fiber was installed in the pipeline. Under the natural flow of wastewater, the fiber was bent by mechanical force and generated piezoelectric polarization, thus enhancing the catalytic performance at the interface between MoS_2_ and carbon fiber, producing hydroxyl radicals (·OH) and an electrochemical reaction to decompose organic matter in wastewater. An amount of 1 L of degraded 10 ppm methyl blue was completely decomposed within 40 min. Ingh et al. [198] calcinated the prepared ZnO/PAN NFs to obtain mesoporous ZnO NFs. When the naphthalene and anthracene dyes were degraded, the contact area between ZnO and them was larger and the reaction rate was faster, which effectively improved the photocatalytic efficiency.

It is promising to enhance the piezoelectric properties of polymer NFs by adjusting the electrospinning process parameters to control the nanofiber morphology and electroactive phase content, which is very promising in piezoelectric catalytic applications. Many catalysts have good catalytic performance [199]. In particular, piezoelectric polymer NFs have good biocompatibility, non-toxicity, and flexibility, which makes them outperform many ceramic materials for certain environmental remediation. NaNbO_3_ is a typical single-component catalyst. Under the irradiation of ultraviolet light, NaNbO_3_ nanorods produce electrons and holes and then produce hydroxyl radicals and superoxide radicals to degrade organic matter. When ultrasonic vibration gives NaNbO_3_ mechanical strain, it produces polarization due to piezoelectric effect. It is important to reduce the recombination of photogenerated charge carriers and improve the degradation efficiency [200]. In order to combine the functions of each component more effectively, researchers also constructed a core–shell-structured piezoelectric photocatalyst to prepare a PZT/TiO_2_ composite structure, and synthesized a core–shell-configuration piezoelectric catalyst nanoparticle by coating TiO_2_ with PZT microspheres. PZT can generate a built-in electric field to effectively prevent the electron–hole pair recombination generated by TiO_2_, thus improving the catalytic capacity [201]. Zhang et al. [14] studied the photocatalytic activity of two MOFs. The dipole moment of Zr/HF–oxo clusters was calculated by density functional theory (DFT), and the dipole moment of the HF–oxo cluster was calculated to be 2.940 Debye. This was much higher than the dipole moment of the Zr–oxo cluster (1.739 Debye) as shown in the Figure 15(1), so the distance between the positive and negative charge centers of UIO-66-NH_2_ (Hf) shows a larger change under mechanical stretch or strain along the asymmetric direction, leading to stronger spontaneous polarization and a larger piezoelectric response. The piezoelectric performance test results showed that the electrical activity of the HF-based UO-66-NH_2_ is 2.2 times that of the Zr-based, and MOF can catalyze hydrogen production under both light and ultrasonic mechanical vibration and can be loaded on the NFs to improve their photocatalytic performance.

### 5.3. Energy Conversion

In recent years, with the gradual depletion of primary energy such as traditional fossil fuels and the improvement of environmental awareness, the demand for clean energy and renewable energy has gradually increased. Compared with solar energy, tidal energy, wind energy, geothermal energy, and other energy sources that require large-scale energy conversion devices, the micro-electromechanical conversion system using piezoelectric materials can be more convenient for some applications and can also collect mechanical energy and convert it into electric energy utilizing small mechanical forces such as human kinematics and micro-vibrations.

**Figure 16:**
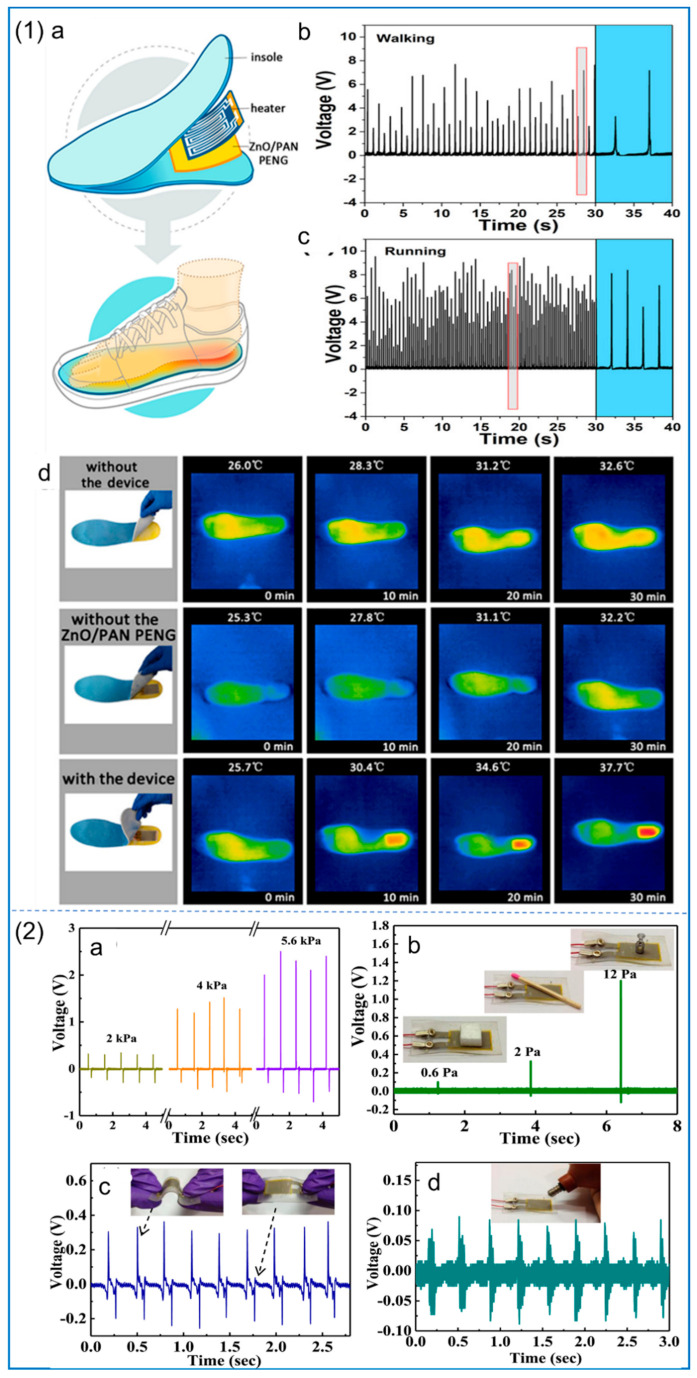
(**1**) The electric energy made by PNG is used to drive the electric heating plate. (**a**) Schematic diagram of self-heating insole. Digital photo and infrared image of the output voltage generated by PNG during (**b**) walking and (**c**) running, the blue inset is a partial enlargement of the red box, (**d**) insole. Reprinted with permission from Ref. [146], copyright 2020, American Chemical Society. (**2**) (**a**) Plot of voltage response as a function of time for different applied pressures under finger impingement. (**b**) Output voltage response of PNG with polystyrene, matchstick, and weight placed at the top. Piezo−response plots for loading and unloading cycles under (**c**) bending and (**d**) blower pressure. Reprinted with permission from Ref. [203], copyright 2018, Elsevier.

Recently, the electrostatic spinning technique used to prepare NFs used in generators has become a hot spot. Because the electrostatic spinning technology can improve the piezoelectric properties of materials, Zhu et al. [202] use the electrostatic spinning technique to prepare PLLA NFs; this method of preparation of the nanometer carbon fiber’s main chain of electric dipole polarization components can be along the alignment direction. Moreover, the piezoelectric properties of NFs can be affected by controlling the electrospinning parameters, and the piezoelectric properties of polymers can also be improved by adding nanoparticles and surfactants. Sun et al. [146] loaded ZnO nanorods (ZnO NRs) onto PAN to form ZnO/PAN composite NFs, and fabricated PNG to drive the electric heating plate, as shown in Figure 16(1). Moreover, the NFs’ piezoelectric sensitivity and energy harvesting efficiency are about 2.7 times higher than those of pure PAN, and their output power is twice that of ZnO/PVDF composite NFs. This excellent performance is attributed to the improved zigzag conformation in PAN NFs by the addition of ZnO NRs. In the medical field, this piezoelectric composite nanofiber membrane can be used as a power supply device for small medical devices [129]. ZnO/rGO/PVDF–TRFE composite NFs can be used in battery-free cardiac pacemakers. The piezoelectric properties were also increased by the addition of nanoparticles ZnO and rGO. The hydrogen atoms in ZnO, rGO, and PVDF–TRFE interact with fluorine atoms so that the fluorine atoms are arranged in one direction in the main chain to form β phase (TTTT). The pacemaker has demonstrated its excellent performance in animal experiments, and it can collect more energy per heartbeat than human heart pacing. Sultana et al. [203] studied the addition of methyl lead ammonium bromide (MAPbBr) to PVDF in electrospinning to make PNG, and the relevant tests are shown in Figure 16(2). The results show that the content of β phase in PVDF is increased to 91%, and the PNG has high piezoelectric sensitivity and energy harvesting efficiency.

Human kinematics is the main source of piezoelectric energy collected by NFs, which show great potential in self-powered wearable devices, converting mechanical energy into electrical signals through walking, joint bending, sound vibration, and other means. It is promising to prepare these piezoelectric materials into flexible fabrics or wearable items for use in human life. Particularly, some raw natural materials [204,205,206,207] and nanostructures [208,209,210] may play roles in the developments of these piezoelectric materials. 

## 6. Conclusions and Perspectives

Electrospinning technology is a simple and efficient technology for preparing nanofiber films. Various properties of nanofiber films can be well-controlled by adjusting spinning parameters. In particular, the content of piezoelectric phase in piezoelectric polymers can be greatly increased by stretching and electric polarization of the jet, so as to improve their piezoelectric properties. Piezoelectric NFs prepared by electrospinning have better flexibility and biocompatibility than traditional ceramic piezoelectric materials. By adding different additives to piezoelectric materials with electrospinning technology to produce piezoelectric properties of nanometer fiber membranes, we observe increases in piezoelectric performance improvement factors, including piezoelectric phase content. Additives themselves have piezoelectric properties and by changing the solution properties in electrospinning, NFs’ piezoelectric properties also change. Many piezoelectric polymer NFs, especially those containing fluorine polymers with good biocompatibility, also have very good piezoelectric constants, but they are biodegradable and their renewable property is lower, so it is necessary to research some natural polymers. By electrospinning, parameters optimized the synthesis of natural polymers and enhanced piezoelectric properties.

1. The high-voltage electric field applied during electrospinning has a significant effect on the in situ polarization and induced piezoelectric phase formation, so this method has a broad prospect in the preparation of piezoelectric materials;
2. By studying ways to change the piezoelectric properties of piezoelectric polymers, it is expected that the piezoelectric properties of piezoelectric polymers can be continuously improved to meet the needs of various applications;
3. Piezoelectric NFs can convert mechanical energy from various small vibrations into electric energy. Because of their good flexibility, piezoelectric NFs have been widely studied in wearable electronic devices;
4. Due to their good biocompatibility, piezoelectric polymers have broad application prospects in the biomedical field, such as in power supply for tissue engineering scaffolds, cardiac pacemakers, and other small internal medical devices, which is promising for clinical treatment after a lot of research;
5. Piezoelectric NF membranes prepared by electrospinning also have important applications in the field of piezoelectric photocatalysis. The piezoelectric effect can generate a built-in electric field inside the material to inhibit the composition of photogenerated charge carriers, improve the carrier life, and enhance the catalytic efficiency. Some single-component piezoelectric photocatalysts are known to have excellent catalytic performance. It is expected that piezoelectric photocatalysts with better catalytic performance can be obtained by combining them with electrospinning;
6. Electrospinning is moving forward rapidly from single-fluid processes to coaxial, triaxial, side-by-side, and tri-layer side-by-side processes for generating all kinds of complicated nanostructures [211,212,213,214,215]. With PVDF as a filament-forming polymeric matrix, more and more excellent piezoelectric nanomaterials are predicted to be witnessed in future.

## Figures and Tables

**Table 1:** Piezoelectric constants and crystal structures of common piezoelectric materials.

Category	Materials	Piezoelectric Charge Constant, d_ij_ (pC/N)		Crystal Structure
		Bulk	Nanofiber	
Ceramic materials	PbNb_2_O_6_	d_33_ = 57 [42]	—	Orthorhombic (tungsten bronze)
	PbTiO_3_	d_33_ = 97 [43]	—	Tetragonal
	BaTiO_3_	d_33_ = 95 [44]	—	Tetragonal (perovskite)
	PBLN	d_33_ = 350 [45]	—	Orthorhombic
	Li_2_B_4_O_7_	d_33_ = 8.76 [46]	—	Tetragonal
	PZT	d_33_ = 223 [47]	—	Tetragonal (perovskite)
	ZnO	d_33_ = 12.3 [48]	—	Hexagonal (wurtzite)
Polymer materials	PVDF	d_31_ = 23 [49], d_33_ = 15 [50,51]	d_33_ = 57.6 [52]	α, β, γ, δ, σ-phase
	PLLA	d_14_ = 6.0 [49]	d_33_ = 3 ± 1 [53]	α, β, δ-phase
	PHBV	d_33_ = 0.43 [54]	d_33_ = 0.7 ± 0.5 [55]	α, β
	PAN	d_31_ = 0.6 [56]	d_33_ = 39 [57], 1.5 [58]	Zigzag
	Nylon-11	d_33_ = 6.5 [59], d_31_ = 14 [60]	—	α, α’, β, β’
	Collagen	d_14_ = 12 [61], d_33_ = 2 [62]	d_15_ = 1 [63]	Triple helix
	Cellulose	d_25_ = 2.1 [64]	d_33_ = 31 [65]	I_α_ (triclinic), I_β_ (monoclinic)
	Chitin	d_33_ = 9.49 [66]	—	α, β, γ-phase
	Chitosan	d_33_ = 4.4 [67], d_31_ = 10 [68]	—	Double helix

**Table 2:** The influence of Various additives to PVDF.

Category	Material	Influence	Addition (wt%)	Average Diameter (μm)	Peak Voltage (V)	Modulus of Elasticity (MP)	Application
Inorganic piezoelectric material	PZT [107]	Enhanced the mechanical and piezoelectric properties of the fiber membrane	20.0	370	—	2053	Piezoelectric pressure sensor
	BaTiO_3_ [108]	Enhanced piezoelectric performance	16.0	0.200	0.48	—	Smart textiles and biomedical devices
	ZnO [109]	Improved the crystal structure and improved the electrical output	15.0	0.757	1.10	—	Self-powering of microelectronics clothing
	G-ZnO [110]	Reduced average diameter, increased the β phase, and improved piezoelectric performance	1.0	0.087	0.84	—	Wearable piezoelectric nanogenerators
Conductive particles	AgNO_3_ [111]	Conductivity increased	0.3	0.201	2.00	4047	Nanoelectronics, and energy harvesting
	FeCl_3_·6H_2_O [111]	Conductivity increased, increased the β phase	0.8	0.254	4.80	5621	
	Modified graphene [111]	Conductivity increased, increased the β phase, improved the mechanical properties	1.0	0.296	1.80	6583	
	Carbon nanotubes [112]	Increased specific surface area, increased the β-phase content, and improved the piezoelectric properties	18	0.138	—	—	Efficient sound absorbers
	BNNs [113]	Enhanced the triboelectric effect	2.0	0.007	0.25	—	Human motion detection
MOF	Uio-66(Zr) [114]	Increased the β phase, its own piezoelectricity	5.0	0.053	0.57	1100	Flexible energy convertors, biomedical monitoring
	[CdI_2_−INH=CMe_2_] [115]	Increased the β phase, controllable porous structure	1.0	—	12.00	—	Real-time wearable medical devices
Organic additives	DDAC [116]	Increased the β phase	8.0	0.09	—	—	Artificial intelligence

## Data Availability

The data supporting the findings of this manuscript are available from the corresponding authors upon reasonable request.

## Abbreviations

PBLN	Barium lead niobate
PZT	Lead zirconate titanate
PVDF	Polyvinylidene fluoride
PLLA	Poly-L-lactic acid
PHBV	Poly(3-hydroxybutyrate-co-3-hydroxyvalerate)
PAN	Polyacrylonitrile
Nylon-11	Poly-ω-aminoundecanoyl
PZT	Lead zirconate titanate
G–ZnO	Graphene–zinc oxide
BNNs	Boron nitride nanosheets
DDAC	Dioctadecyl dimethyl ammonium chloride
DMF	Dimethylformamide
ACE	Acetone
DMAc	Dimethylacetamide
MOF	Metal organic framework
BNNs	Nitride nanosheets
CNTs	Carbon nanotubes
KNN	Potassium sodium niobate
C-PNG	Composite fiber-based piezoelectric nanogenerator
DDAC	Dioctadecyl dimethyl ammonium chloride
PVDF–TRFE	Poly (vinylidene fluoride–trifluoride)
PVDF–TFE	Poly (vinylidene fluoride–tetrafluoride)
PVDF–HFP	Poly (vinylidene fluoride–hexafluoride)
PEO	Polyethylene oxide
rGO	Reduced graphene oxide
PNG	Piezoelectric nanogenerators
BCZT	Barium calcium zirconia titanate
PLLA	Poly-l-lactic acid
PHB	Poly (3-hydroxybutyric acid)
HAP	Hydroxyapatite
BTO	Barium strontium titanate
MWCNTs	Multi-walled CNTs
PANI	Polyaniline
PCL	Polycaprolactone
TFA	Trifluoroacetic acid
DEX	Dexamethasone
MSCS	Mesenchymal stem cell
rhBMP-2	Recombinant human bone morphogenetic protein-2
PPy	Polypyrrole
AAO	Anodized aluminum oxide
RhB	Rhodamine B
CB	Conduction band
HOMO	Highest-occupied molecular orbital
VB	Valence band
LUMO	Lowest-unoccupied orbital
·OH	Hydroxyl radical
DFT	Density functional theory
ZnO NRs	ZnO nanorod
MAPbBr	Methyl lead ammonium bromide
